# Nigericin Promotes NLRP3-Independent Bacterial Killing in Macrophages

**DOI:** 10.3389/fimmu.2019.02296

**Published:** 2019-10-01

**Authors:** Heather Armstrong, Michael Bording-Jorgensen, Richard Chan, Eytan Wine

**Affiliations:** ^1^Department of Pediatrics, University of Alberta, Edmonton, AB, Canada; ^2^Centre of Excellence for Gastrointestinal Inflammation and Immunity Research (CEGIIR), University of Alberta, Edmonton, AB, Canada; ^3^Department of Physiology, University of Alberta, Edmonton, AB, Canada

**Keywords:** NLRP3, inflammasome, inflammatory bowel diseases, *Citrobacter rodentium*, nigericin

## Abstract

Altered microbiota has been associated with a number of diseases, including inflammatory bowel diseases, diabetes, and cancer. This dysregulation is thought to relate the host inflammatory response to enteric pathogens. Macrophages play a key role in host response to microbes and are involved in bacterial killing and clearance. This process is partially mediated through the potassium efflux-dependent, cytosolic, PYCARD-containing inflammasome protein complex. Surprisingly, we discovered an alternative mechanism for bacterial killing, independent of the NLRP3 inflammasome/PYCARD. Using the NLRP3 inflammasome-deficient Raw 264.7 and PYCARD-deficient J77 macrophages, which both lack PYCARD, we found that the potassium efflux activator nigericin enhances bacterial killing. Macrophage response to nigericin was examined by RT gene profiling and subsequent qPCR, which demonstrated altered expression of a series of genes involved in the IL-18 bacterial killing pathway. Based on our results we propose a model of bacterial killing, unrelated to NLRP3 inflammasome activation in macrophage cells. Improving understanding of the molecular pathways driving bacterial clearance within macrophage cells will aid in the development of novel immune-targeted therapeutics in a number of diseases.

## Introduction

Dysbiosis or altered microbiota, is characteristic of a number of diseases including inflammatory bowel diseases (IBD), obesity, diabetes, autism, and cancer (1), likely mediating a dysregulation of the host immune response (2–4). Detailed analysis of the intestinal inflammatory response to enteric pathogens may be key to understanding disruption of homeostasis and disease progression and discovering novel therapies.

Interestingly, bacterial DNA can stimulate activation of host innate immune cells through recognition of pattern-recognition molecules (PRMs) by microbe-associated molecular patterns (MAMPs) (5–7). PRMs are located on or within innate immune cells such as dendritic cells, macrophages, and neutrophils, along with epithelial cells. Examples of transmembrane PRMs are toll-like receptors (TLRs) and C-type lectins (CLRs); cytosolic PRMs include members of the NOD (Nucleotide Oligomerization Domain)-like receptor (NLR) family and DNA sensing DAI and AIM2 complexes (8). Macrophages are especially pivotal for a functional immune response as they are involved in bacterial killing and clearance by engulfing and eradicating invading pathogens (9). We have previously demonstrated that bacterial clearance by macrophages can be improved through the stimulation of the multiprotein complex known as the inflammasome, through extracellular ATP (10). Increased bacterial clearance from ATP activation was associated with a reduction of pro-inflammatory cytokines after clearance, suggesting a potential mechanism for the link between increased susceptibility to IBD and inflammasome dysfunction (10, 11).

A number of inflammasomes have been identified, including NLRP1, NLRP2, NLRP3, and NLRC4 (12). The most well-characterized inflammasome is NLRP3, in which NLRP3 forms a complex with PYCARD [also known as the adapter protein apoptosis-associated speck-like protein (ASC)] and procaspase-1 (13, 14). Assembly of this complex results in self-cleavage of procaspase-1 to active caspase-1 (15), leading to the activation of the proinflammatory cytokines interleukin (IL)-1β and IL-18 (8, 16–18). NLRP3 activation involves a two-step process including a priming signal, which includes recognition of MAMPs (e.g., bacteria or lipopolysaccharide) by cell surface TLRs, followed by a second step involving NLRP3 complex assembly and activation (12, 19–22). Activation of the inflammasome is driven by agonists such as adenosine triphosphate (ATP), which indirectly regulates NLRP3 activity via stimulation of P2X_7_ nonselective K^+^ or Ca^2+^ channel receptors (8).

While inflammasome modulators such as Nigericin are described as NLRP3-specific (23), their indirect effects led us to hypothesize that they may be able to regulate inflammation and bacterial killing via inflammasome-independent mechanisms. Nigericin is a toxin derived from *Streptomyces hygroscopicus*. It is described as a potassium ionophore, which facilitates H^+^/K^+^ anti-port across cell membranes, thereby activating NLRP3 by causing potassium efflux. In this *in vitro* study, we examined the effects nigericin, an NLRP3 activator, on Raw 264.7 macrophages, which lack PYCARD/ASC and therefore are incapable of NLRP3 activation. As Raw 264.7 cells are a murine cell culture model, we implemented the mouse pathogen *Citrobacter rodentium* for our *in vitro* experiments. *C. rodentium* shares many virulence factors such as formation of attaching/effacing (A/E) lesions with the commonly studied human intestinal *Escherichia coli* pathogenic strains Enterohaemorrhagic *E. coli* (EHEC) and Enteropathogenic *E. coli* (EPEC), resulting in transmissible colonic hyperplasia, colitis, and bloody diarrhea (24, 25). Our results demonstrate that the NLRP3 agonist, nigericin, promotes killing of *C. rodentium*, and induction of an inflammatory response through pathways unassociated with the NLRP3 inflammasome complex, independent of ASC. Understanding this previously unrecognized mechanism of bacterial clearance within a macrophage cell system will aid in the ability to identify new methods of altering the immune response in a number of human diseases driven by altered host-microbe interactions, such as IBD.

## Materials and Methods

### Cell Culture

Murine macrophage cell line Raw 264.7 were obtained from the American Type Culture Collection (ATCC; Maryland, United States) and cultured in Dulbecco's Modified Eagle Medium (DMEM), 10% fetal bovine serum (FBS), 5% penicillin/streptomycin and incubated (37°C, 5% CO_2_) until confluent for a maximum of 20 passages.

Murine macrophage cell line J774A.1 were obtained from the ATCC and cultured in DMEM containing 10% FBS for a maximum of 25 passages.

*C. rodentium* (strain DBS100) was provided as a gift by Dr. Philip Sherman (University of Toronto) and cultured aerobically in lysogeny broth (LB) at 37°C.

### Standard Curves

Raw 264.7 cells were seeded at 1.5 × 10^5^ cells per well in a 24 well plate and cultured overnight at 37°C and 5% CO_2_. Cells were treated as indicated below with ATP (Sigma), Ac-Tyr-Val-Ala-Asp-Chloromethylketone (YVAD; Sigma), nigericin (Sigma), or Gentamicin (Fischer Scientific) to determine associated toxicity or growth inhibition. Cells were manually counted using a haemocytometer and trypan blue exclusion technique to determine cell number at the indicated time points.

*C. rodentium* liquid LB cultures were seeded and grown overnight at 37°C. Growth density was measured at OD_600_ on a spectrophotometer and serial dilutions were cultured on LB agar overnight. Colony counts were performed on each plate and used to calculate colony forming units (CFU)/mL from each dilution.

*C. rodentium* liquid LB cultures treated with nigericin (20 μM) were seeded and grown overnight at 37°C. Cells were diluted to an OD_600_ of 0.1 (representing 8 × 10^7^ CFUs/mL) and treated with nigericin at time 0 h. Growth density was measured at OD_600_ on a spectrophotometer at indicated time points and compared to untreated growth.

### siRNA

Raw 264.7 or J77 cells were plated (8 × 10^4^ cells per well) in 24 well plates overnight at 37°C and 5% CO_2_ to adhere. Cells were then treated with siRNA as indicated or scramble siRNA control using Lipofectamine 2,000 by manufacturer's instructions. Hundred pmol of siRNA was used to efficiently knock down p38 (Dharmacon; AUGAGGAGAUGACCGGAUA) and IFNγ (Dharmacon; UCGAAUGAGUCAGGUAGUA), or 50 pmol siRNA phosphorylated ASC as demonstrated by western blotting following 48 h siRNA.

### Gentamicin Protection Assay

**Figure 1:**
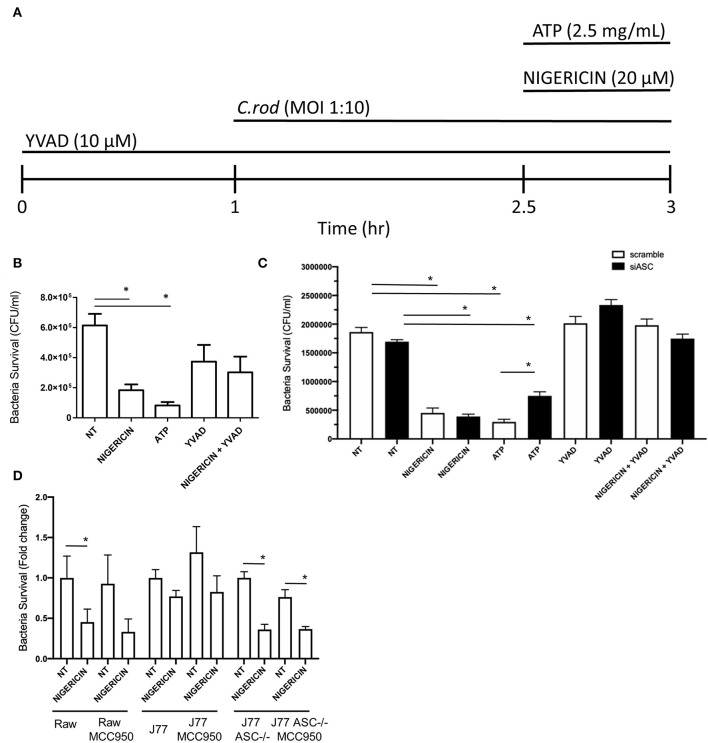
Nigericin improves bacterial killing of *C. rodentium* independent from the NLRP3 inflammasome. **(A)** Raw264.7, J77^ASC−/−^, or J77^scramble^, cells were first treated with YVAD as indicated for a total of 3 h. Inoculation of cell culture with *C. rodentium* (MOI 1:10) occurred 1 h after initial addition of YVAD. 1.5 h after *C. rodentium* inoculation, Nigericin (20 μM) or ATP control (25 mg/mL) was added to the culture media. Gentamicin (25 ug/mL) was used to eliminate non-invasive bacteria in **(B)** Raw264.7 cells or **(C)** J77^ASC−/−^, and J77^scramble^, and **(D)** cells treated with or without MCC950 (1 μM). Cell lysates were collected and grown on LB agar overnight and colonies were manually counted and expressed as CFU/mL. Values represent mean ± SEM between 3 independent experiments; *p < 0.05. YVAD (Ac-Tyr-Val-Ala-Asp-Chloromethylketone); MOI (multiplicity of infection); CFU (colony forming units).

Raw 264.7 or J77 cells were seeded into a 24-well plate overnight at a density of 2 × 10^5^ cells per well. Medium was removed and replaced with serum-free DMEM prior to beginning treatment. Cells were then treated with either YVAD (10 μM) and/or nigericin (20 μM), as detailed in Figure 1A. Treatment with YVAD occurred 1 h prior to *C. rodentium* inoculation and proceeded for 3 h total. Raw 264.7 macrophages were inoculated with *C. rodentium* [multiplicity of infection (MOI) 1:10] 1 h after YVAD (when appropriate) for 2 h total. Nigericin treatment or ATP control (2.5 mM) was added 1.5 h post-infection and proceeded for 30 min total. Both non-infected and infected, no treatment (NT) controls were included. Medium was removed and 20 μg/mL of gentamicin was added to each well for 3 h to kill any bacteria not engulfed by the macrophages (gentamicin does not penetrate the eukaryotic cell membrane). Cells were lysed with 1% TritonX-100 for 30 min to enumerate intracellular/engulfed bacteria. Lysates were plated on LB agar in serial dilutions and colonies were manually counted the following day to calculate intracellular CFU/mL. Results were repeated again following pre-treatment with siRNA directed toward p38, Interferon gamma (IFNγ), both p38 and IFNγ, or ASC as indicated. Further results were repeated using the NLRP3 inhibitor MCC950 (Invivogen) at 1 μM for 3 h prior to infection with *C. rodentium* and nigericin treatment as previously performed.

### ELISA

Raw 264.7 or J77 cells were seeded at a density of 2 × 10^5^ cells per well into a 24-well plate and grown overnight. Medium was removed and replaced with serum-free DMEM prior to beginning treatment. J77 cells were included as a positive active-NLRP3 control. Cells were then treated with YVAD (10 μM) 1 h prior to *C. rodentium* inoculation and proceeded for 3 h total, as above. Macrophage cells were inoculated with *C. rodentium* (MOI 1:10) for 2 h total. KCl (45 mM) was added at the time of infection. Nigericin (20 μM) treatment or ATP control (2.5 mM) was added 1.5 h post-infection and proceeded for 30 min total. Both non-infected and no treatment (NT) controls were included. Supernatants were collected and centrifuged at 14,000 g for 10 min to remove any bacteria, cells, or debris. Cell lysates were collected by applying 150 μl radioimmunoprecipitation assay (RIPA) buffer to each well. Cells were removed by aggressive pipetting and centrifuged at 14,000 g for 10 min to remove cell debris. Protease inhibitor (1:100; Sigma Aldrich) was added to the supernatant and secreted IL-1β, IL-18, or p38 was measured using an ELISA following manufacturers protocol (R&D Systems). Limit of detection for these ELISA kits is 15.6 pg/mL.

### Western Blot

Lysates were collected by applying 150 μl RIPA buffer to each well. Cells were removed by aggressive pipetting and centrifuged at 14,000 g for 10 min to remove cell debris. Total protein levels were determined by Bradford assay and 25 μg total protein was loaded per sample into a BioRad 12% precast TGX acrylamide gel. Nitrocellulose membranes were probed for Mapk11 (Abcam; ab80116), IFNγ (Abcam; ab133566), lamin (Proteintech; 66095-1), ASC (Abcam; ab70627), and actin (Santa Cruz; sc-1616). Membranes were imaged using the BioRad ChemiDoc Gell Imaging System.

### Phagocytosis Assay

Raw 264.7 cells were prepared as described for the gentamicin protection assay. In order to dissociate phagocytosis from bacterial invasion of Raw 264.7 cells, we used inert beads to measure effects on phagocytosis alone; 1:10 (Raw 264.7 cells: beads) of 2 μm conjugated beads (Polysciences Inc.) were added to the culture for 2 h. Cells were treated as previously described and fixed using 4% paraformaldehyde, then stained with β-actin primary antibody (1:40; Abcam) and DAPI (1:1,000; Thermo Fischer). Slides were examined using an Olympus IX-81 microscope with a Yokagawa spinning disk confocal head, 60X oil immersion lens with a 1.42 numerical aperture, and a Hamamatsu EMCCD camera. Images were taken with equal exposure time without saturation and analyzed with Volocity imaging software (PerkinElmer). Illustrations were formatted, for noise reduction and increased sharpness, using Image J (National Institute of Health, Maryland, USA). Phagocytosis was measured by the number of beads inside the macrophages divided by the total number of macrophages in each randomly selected image. Ten photos of each treatment were taken from duplicate repeats.

### RNA Isolation and Gene Expression Analysis

Raw 264.7 cells were cultured overnight prior to treatment as indicated. RNA was isolated using 1 mL trizol as previously described (26). Reverse transcription was performed using iScript master mix. Inflammatory cytokines and receptors RT^2^ profile array (QIAGEN) was run using a BioRad CFX96 thermocycler according to manufacturer's instructions (40 cycles of 95°C to 60°C). RT-qPCR was performed as previously described (26) to validate findings using the primers highlighted in Table 1. Both biological and technical replicates were performed on all reactions using β-Actin and Rer1 as housekeeping genes. Data were analyzed using CFX Manager Software Version 3.0 (Bio-Rad Laboratories, Inc.).

**Table 1:** Primer sequences used in RT-qPCR.

**PRIMER**	**Forward**	**Reverse**
IFN-γ	TTCTTCAGCAACAGCAAGGC	CCTTTTCCTCAGCGACGACT
IL-12	GATGACATGGTGAAGACGGC	AACTACTACTGGGACACGGA
Ciita	TGCAGGCGACCAGGAGAGACA	GAAGCTGGGCACCTCAAAGAT
P38	CAGAAGGACCTCAGCAGTGTCT	GTACTGGCTGAAGTATGCGTGG
Cd40lg	GAACTGTGAGGAGATGAGAAGGC	TGGCTTCGCTTACAACGTGTGC
tab2	CATTCAGCATCTCACAGACCCG	CTTTGAAGCCGTTCCATCCTGG
Nlrp4e	CTCTGTCCAAGGCTTTGTGCCA	TGGGTCAAGGTTTTGTTCCGCC
Tnfsf11	GTGAAGACACACTACCTGACTCC	GCCACATCCAACCATGAGCCTT
Nlrp12	GGAAGAGACAGCAGACTCGAGAATCTTTTTCATC	GATGAAAAAGATTCTCCTCTCTGCTGTCTCTTCC
NLRP3	TGCTCTTCACTGCTATCAAGCCCT	ACAAGCCTTTGCTCCAGACCCTAT
IL-10	ATAACTGCACCCACTTCCCA	GGGCATCACTTCTACCAGGT
IL-1β	TGGAAAAGCGGTTTGTCT	ATAAATAGGTAAGTGGTTGCC
TNFα	ATGAGCACAGAAAGCATGA	AGTAGACAGAAGAGCGTGGT
β-Actin	GGCTGTATTCCCCTCCATCG	CCAGTTGGTAACAATGCCATGT
Rer1	GCCTTGGGAATTTACCACCT	CTTCGAATGAAGGGACGAAA

### Statistical Analysis

Groups were compared using paired Student's *t*-test (two-tailed) analysis in Microsoft Excel or evaluated by the Student's unpaired *t*-test with Welch's correction using GraphPad Prism 4.0 (GraphPad Software, La Jolla, USA). A *P* < 0.05 was considered as significant in all cases.

## Results

### Optimization of Bacterial Growth and Assessment of Cell Toxicity

Response of Raw 264.7 cells to select non-toxic doses of ATP, YVAD, nigericin, and gentamicin was examined by culture growth curves (Supplementary Figure 1). Raw 264.7 cells were seeded at 1 × 10^5^ cell per well in 24 well-plates and grown overnight before beginning treatment at indicated doses. Effects of each treatment on cell proliferation and death was calculated by manual counting at indicated time points using a haemocytometer (expressed as number of cells/well) and trypan blue exclusion (shown as % of cell death). The following doses with minimal effect on cell growth or survival (similar to dose zero) were selected for further study: 2.5 mg/mL ATP, 10 μM YVAD, 20 μM nigericin, and 20 μM gentamicin.

Growth rates of *C. rodentium* in LB medium in overnight culture was evaluated by standard curve based on OD_600_ readings performed at 24 h post inoculation, compared to manual colony counts performed on serial dilutions of culture grown on LB plates (Supplementary Figure 2A). A trend line was fitted to the scatter plot and followed the formula [y = 2.7537x + 0.374], which was then used to determine the concentration of *C. rodentium* within culture, based on the optical density.

Given the potential toxicity of high doses of Gentamicin to Raw 264.7 cells, we optimized treatment duration. To examine the effects of gentamicin on microbial killing of *C. rodentium* cultured with Raw 264.7 cells, overnight cultures were prepared and infected as indicated in the methods section. To ensure the selected dose of gentamicin (25 μg/mL) resulted in effective microbial killing of extracellular *C. rodentium*, we plated culture supernatant on LB agar prior to lysing cells (data not shown). No growth of *C. rodentium* was found on LB agar following 24 h incubation. Cells were treated with gentamicin and lysed at 2, 4, 6, and 24 h and culture lysates were plated on LB agar. Following overnight growth, colonies were manually counted and expressed as CFU/mL (Supplementary Figure 2B). Results demonstrated that CFU/mL decreased by ~75% between 2 and 4 h. While there was no significant change in CFU/mL between 4 and 6 h, a further reduction in CFU/mL occurred between 6 and 24 h. Subsequent experiments utilized gentamicin for 2 and/or 4 h to ensure that only *C. rodentium* cultures taken up by macrophages and not extracellular microbes were being examined.

### Microbial Killing of *C. rodentium* in Raw 264.7 Is Increased by Nigericin

To examine the effects of nigericin on bacterial killing, Raw 264.7 cells were treated with nigericin (20 μM; 30 min), ATP (2.5 mM; 30 min), or Ac-YVAD-cmk (10 μM; 3 h; Sigma), in the presence of *C. rodentium* as indicated (Figure 1A). Raw 264.7 cells were inoculated with *C. rodentium* without any treatment (NT) as a negative control, or with ATP as a positive control (Figure 1A). Nigericin treatment of Raw 264.7 cells cultured with *C. rodentium* resulted in ~70% reduction in CFU/mL compared to control not treated (NT) cultures (*p* < 0.05; Figure 1B). ATP led to a similar reduction in bacterial invasion compared to NT (*p* < 0.01). No significant change was found between YVAD and NT (*p* > 0.1) or nigericin+YVAD and NT (*p* > 0.1), suggesting that addition of YVAD (caspase-1 inhibition) was able to counteract the bacterial killing effects of nigericin. Results were repeated in J77 cells following siRNA knock down of ASC (Figure 1C) and demonstrated equivalent findings. To ensure the effects of nigericin were not dependent on NLRP3 we repeated these experiments in all cell lines with and without nigericin, and similarly, with and without the NLRP3 inhibitor MCC950. Results demonstrated that MCC950 did not alter bacterial killing in Raw264.7 or J77^ASC−/−^ cell lines in either NT or nigericin treated, *C. rodentium* infected cells (Figure 1D). Interestingly, in J77 cells, MCC950 did not affect bacterial killing in nigericin treated cells but reduced the bacterial killing in NT infected cells, suggesting that while NLRP3 clearly plays a role in bacterial killing, nigericin's effects are also seen through alternate pathways.

**Figure 2:**
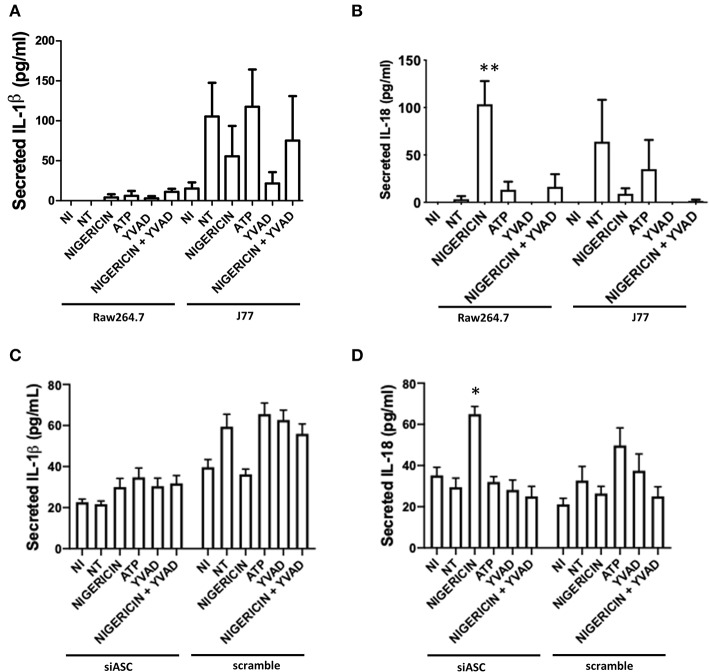
Secretion of IL-18 and not IL-1β, is involved in bacterial killing. Raw264.7 cells or control J77 cells were treated as indicated and inoculated with *C. rodentium* (MOI = 1:10). Supernatants were collected and run on ELISA for **(A)** IL-1β and **(B)** IL-18. J77 cells were then transfected with siASC or scramble control and examined for secretion of **(C)** IL-1β and **(D)** IL-18. Samples were compared to non-infected control (NI) cells which received no treatment or infection. Values represent mean ± SEM between 3 independent experiments; *p < 0.05, **p < 0.01. YVAD (Ac-Tyr-Val-Ala-Asp-Chloromethylketone); MOI (multiplicity of infection).

To test whether microbial killing occurred through activation of the NLRP3 inflammasome, Raw 264.7 and J77 cells were treated as indicated (Figure 1A) and supernatants were collected for ELISA to examine levels of secreted IL-1β, the main outcome of inflammasome activation. J77 macrophage cells were used as a positive control as this cell line expresses ASC (27). Only J77-activated macrophages displayed secretion of IL-1β, indicating that microbial killing was independent of NLRP3 in Raw 264.7 macrophages (Figure 2A). We further analyzed secretion of IL-18, an alternate pathway involved in bacterial killing (Figure 2B). These results demonstrate that Nigericin treatment increased secretion of IL-18 in Raw 264.7 cells while ATP promoted IL-18 secretion in J77 control cells. Analysis of cell lysates demonstrated that there was no altered expression of total IL-1β or IL-18 (Supplementary Figures 3A,B) as neither RNA or cytosolic levels change yet secretion was altered. Results were replicated in J77^ASC−/−^ cells and demonstrated similar response to treatment compared to ASC-deficient Raw264.7 cells (Figures 2C,D). Knock down of ASC reduced IL-1β secretion compared to scramble control. Furthermore, Nigericin induced IL-18 in J77^ASC−/−^ cells, while scramble control led to secretion of similar levels of IL-18 in response to the indicated treatments as non-transfected J77.

### Nigericin Did Not Directly Reduce Bacterial Growth or Alter Phagocytosis in Raw 264.7 Cells

**Figure 3:**
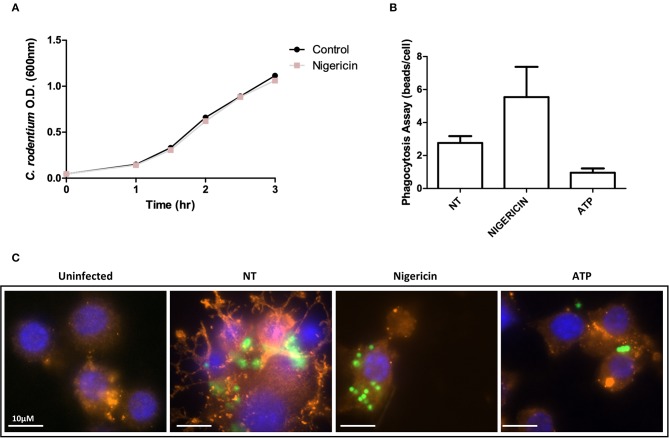
*C. rodentium* growth and phagocytosis of are not altered with nigericin treatment. **(A)** *C. rodentium* were diluted to an initial OD600 of 0.1 and treated with nigericin (20 μM) at time 0 h. OD600 readings were taken at indicated times and compared to non-treated cultures. **(B)** Raw 264.7 cells were cultured with 2 μm conjugated beads (Polysciences Inc.) then treated with nigericin (20 μM) or ATP (2.5 mM) for 30 min. **(C)** Phagocytosis was measured by manual counting. Cells were fixed and stained using DAPI (cell nuclei; blue), Phalloidin (F-actin; red), and Alexa Fluor 488 (*C. rodentium*; green). Cells were imaged using a Leica SP5 confocal microscope at 60x mag.

The effect of nigericin on growth of *C. rodentium* was examined by OD_600_ spectrophotometer readings taken at indicated time points to exclude a direct effect on bacteria (Figure 3A). Nigericin had no effect on the growth rate of *C. rodentium* compared to untreated control over 3 h. Next, the effect of nigericin on Raw 264.7 cell phagocytosis was determined by examining uptake of fluorescein-tagged beads. Fluorescein-tagged beads were used instead of bacteria due to the fact that the bacteria would be killed by the macrophage cells under investigation. These macrophage cells are capable of bacterial killing which is altered in response to drug treatments including nigericin. There was no significant change in phagocytosis between NT, nigericin, or ATP control treated cells (*p* > 0.15; Figures 3B,C). In all samples, it appeared that beads were engulfed by Raw 264.7 cells, suggesting that nigericin impacted bacterial killing but not phagocytosis.

### Nigericin Does Not Alter the Classic NLRP3 Inflammasome Pathway

**Figure 4:**
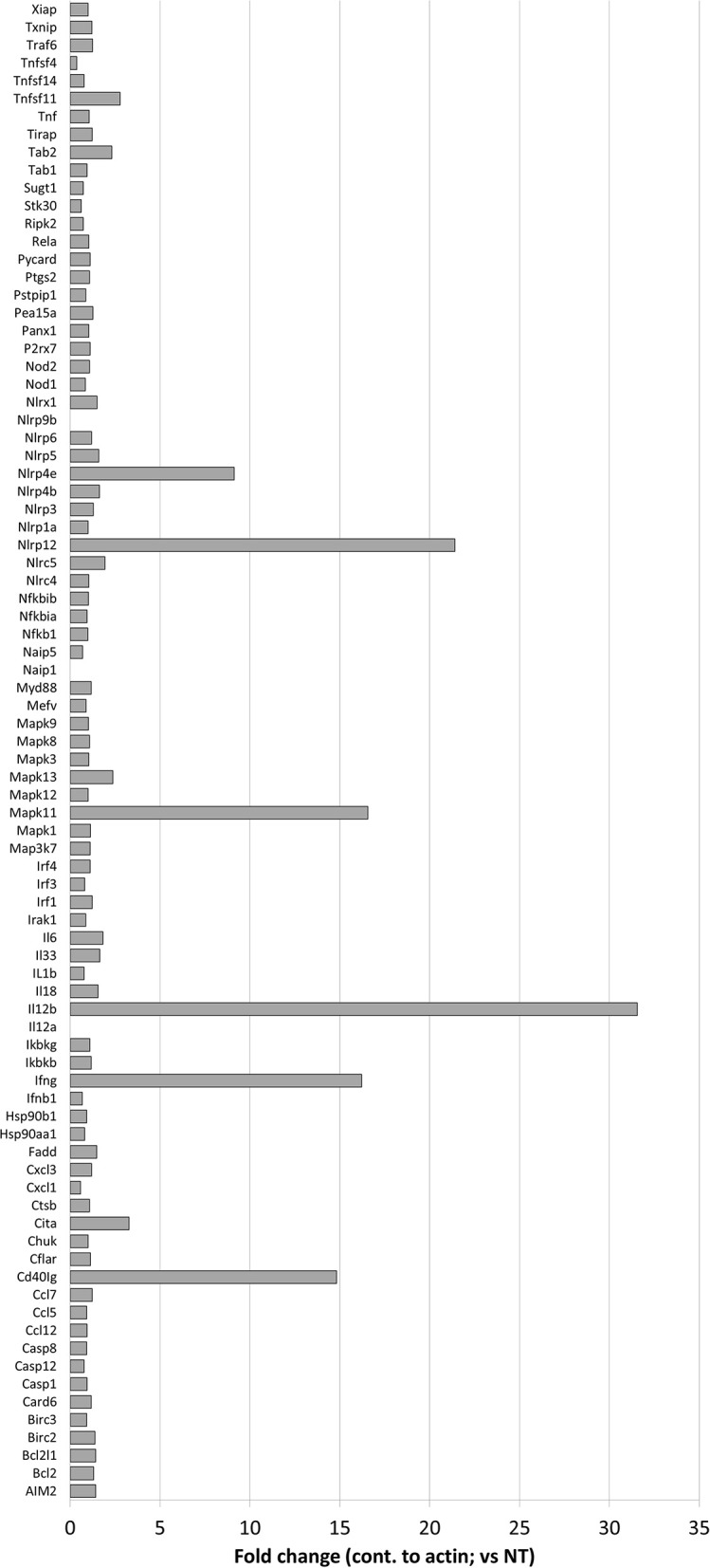
Effects of nigericin on expression of inflammasome-associated genes. QIAGEN inflammasome gene array was performed following the manufacturer's instructions on RNA collected from Raw 264.7 cells treated with nigericin and was compared to untreated cells, following *C. rodentium* infection. Fold changes display nigericin/untreated.

**Figure 5:**
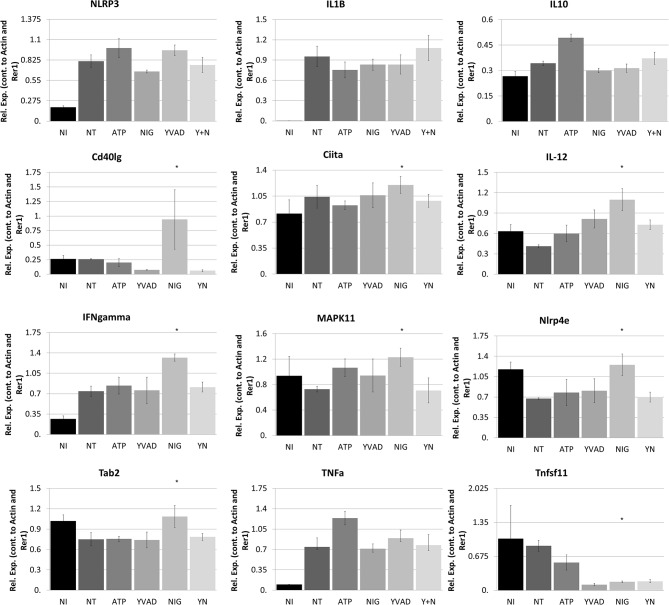
Nigericin results in bacterial killing in Raw 264.7 cells through a mechanism unrelated to the NRLP3 inflammasome. Raw 264.7 cells were first treated with YVAD as indicated for a total of 3 h. Inoculation of Raw 264.7 with *C. rodentium* (MOI 1:10) occurred 1 h after initial addition of YVAD. 1.5 h after *C. rodentium* inoculation, Nigericin (20 μM) or ATP control (2.5 mM) was added to the culture media. Cells were lysed with trizol and RNA extraction and cDNA processing proceeded as described in the methods. qPCR was performed to examine NLRP3, IL-1β, IL-10, Cd40lg, Ciita, IL-12, IFNγ, P38, Nlrp4e, Tab2, TNFα, and Tnfsf11. Samples were controlled against β-actin and Rer1 expression. *p < 0.05.

**Figure 6:**
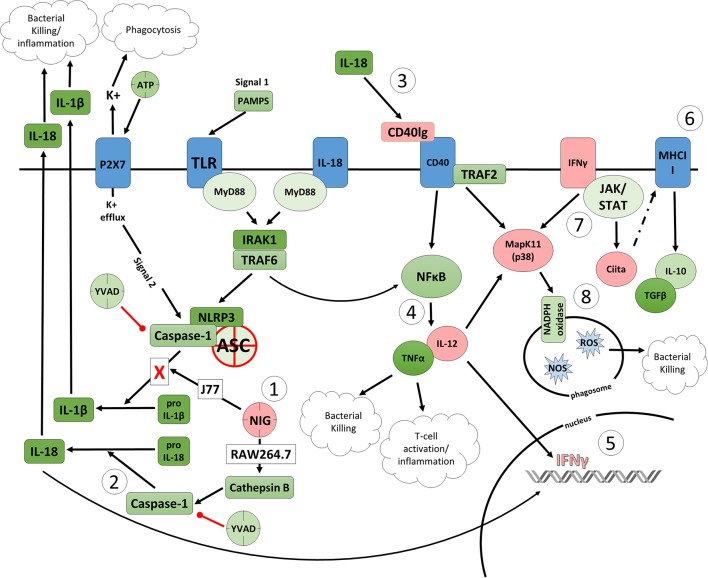
Proposed model of the effects of nigericin on bacterial killing in macrophages deficient of ASC. (1) Caspase-1 is modulated by nigericin via interactions with cathepsin B. Caspase-1 promotes cleavage of the pro-inflammatory cytokine pro-IL-18 (2) to active IL-18, which has been shown to induce upregulation of Cd40lg (3). CD40 is thought to play a key role in inflammatory diseases as hyperactivation of CD40 leads to increased production of pro-inflammatory cytokine, including IL-12 (4). Both IL-12 and IL-18 promote production of IFNγ (5). This cytokine plays a vital role in induction of the MHC class II complex (6), which is critical in macrophage defense against viral, protozoal, and some bacterial infections. IFNγ is an upstream activator of both MAPK and Ciita (7), also known as NLRA. The Ciita/STAT1 pathway is important for MHCII activation and antigen presentation following phagocytosis of bacteria in macrophages, while IFNγ and P38 promote phagocyte and bacterial killing pathways via oxidative burst by increased ROS production (8). Key mediators that were identified by gene array as increased by nigericin are highlighted in pink.

**Figure 7:**
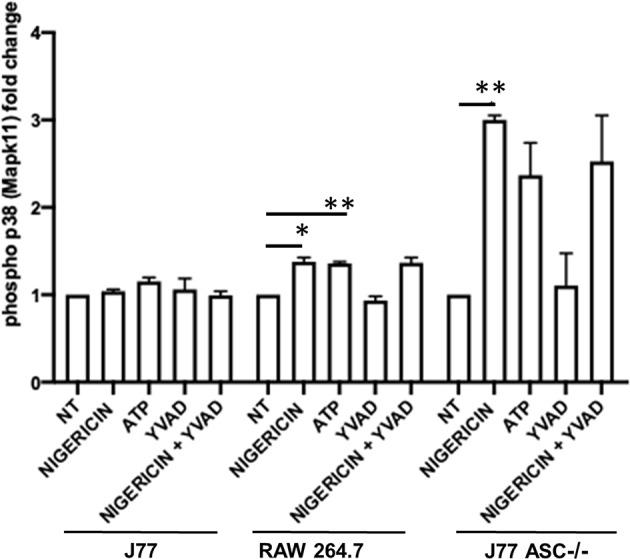
Phosphorylation of p38 (P38) is induced in association with bacterial killing in cells devoid of ASC. Raw264.7 cells, J77^ASC−/−^, or control J77 cells were treated as indicated and inoculated with *C. rodentium* (MOI = 1:10). Protein samples were collected and run on ELISA. Samples were compared to no-treatment control (NT) cells to evaluate the effects of nigericin on p38 phosphorylation. Values represent mean ± SEM between 3 independent experiments; *p < 0.05, **p < 0.01. YVAD (Ac-Tyr-Val-Ala-Asp-Chloromethylketone); MOI (multiplicity of infection).

**Figure 8:**
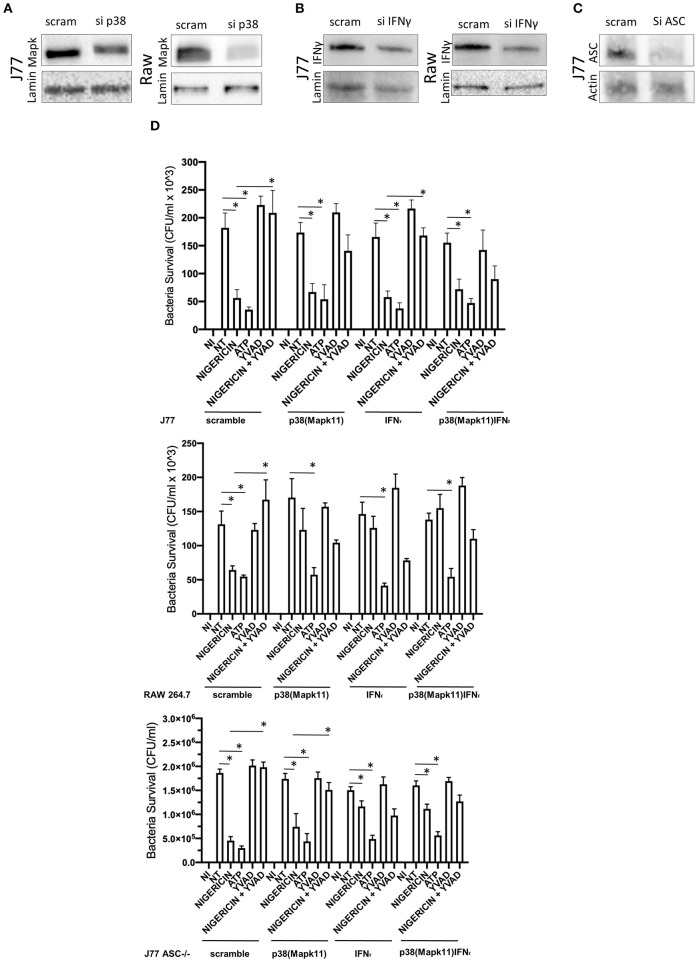
Knockdown of P38 and interferon-gamma ameliorates the effects of nigericin bacterial killing. Dharmacon siRNA directed to P38, interferon-gamma, both P38 and interferon-gamma, or ASC was carried out for 48 h prior to beginning infection and drug treatments. Cells were first treated with YVAD as indicated for a total of 3 h. Inoculation of cells with *C. rodentium* (MOI 1:10) occurred 1 h after initial addition of YVAD. 1.5 h after *C. rodentium* inoculation, Nigericin (20 μM) or ATP control (2.5 mM) was added to the culture media. Cells were lysed with trizol and RNA extraction and cDNA processing proceeded as described in the methods. Knockdown was confirmed by western blot for **(A)** sip38(P38), **(B)** siIFNγ, or **(C)** siASC. **(D)** Gentamicin protection assays were performed to examine bacterial killing in Raw 264.7, J77, and J77^ASC−/−^ cell lines. *p < 0.05.

To better determine the pathways that nigericin alters in Raw 264.7 cells, which may enhance bacterial killing, we performed a gene expression array for inflammatory receptors and cytokines (Figure 4; Supplementary Figure 4). This array demonstrated altered expression of a series of genes, which were then validated by qPCR (Figure 5). Neither NLRP3 nor its downstream target IL-1β resulted in altered expression profile in *C. rodentium* infected cells treated with nigericin when compared to untreated (NT), ATP-treated, YVAD-treated, or nigericin in combination with YVAD. While IL-10 and TNFα expression were elevated slightly in response to ATP, no changes were found in response to nigericin. Nigericin treatment resulted in an increase in Cd40lg, Ciita (NLRA), IL-12, IFNγ, Mapk11 (p38), Nlrp4e, and Tab2 as well as a decrease in Tnfsf11. These results led us to propose an alternate model of bacterial killing in cells devoid of inflammasome activity, such as Raw 264.7 and J77^ASC−/−^ (Figure 6). To examine the effect of nigericin on p38 activity we evaluated the changes in phosphorylated p38 by ELISA of cell lysates (Figure 7). Nigericin increased phosphorylated p38 in Raw264.7 and J77^ASC−/−^ but not in J77 cells. To evaluate the involvement of the IL-18-IFNγ-p38 pathway in bacterial killing, we examined the effects of knock-down of IFNγ or p38 by gentamicin assay (Figure 8). Knockdown of IFNγ, p38, and ASC was evaluated by western blot (Figure 8) following 48 h siRNA treatment for both p38 (Figure 8A; AUGAGGAGAUGACCGGAUA) and IFNγ (Figure 8B; UCGAAUGAGUCAGGUAGUA). As expected, knockdown of IFNγ, p38, or both did not alter the bacterial killing associated with ATP control in either J77 or Raw 264.7 cell lines (Figure 8D). Knockdown did not affect bacterial killing with nigericin in J77 cells. However, knockdown of IFNγ, p38, or both resulted in decreased bacterial killing in Raw 264.7 and J77^ASC−/−^ cells treated with nigericin compared to the scramble siRNA control treated with nigericin (*p* = 0.040), supporting the role of these mediators in the alternative pathway we have identified.

## Discussion

While studies that aim to examine microbial killing by macrophages tend to focus on the inflammasome and its downstream target IL-1β, here we have shown that nigericin, a potassium ionophore (28) and NLRP3 activator, remains capable of increasing bacterial killing in J77^ASC−/−^ and Raw 264.7 cells, independent of the classic NLRP3 inflammasome. Raw 264.7 do not express ASC and are therefore not capable of inflammasome activation or function (27), which we have confirmed in this study. Previous studies have suggested that the effects of nigericin on bacterial killing require the inflammasomes (29), while a limited number of studies have demonstrated that the inflammasome is not essential for bacterial clearance (30), although this was tested in a different setting. Interestingly, while nigericin was able to increase microbial killing in cells lacking ASC, YVAD, a caspase-1 inhibitor that blocks the NLRP3 pathway, was able to partially mitigate or reverse this effect (Figures 1B,C). This suggested that caspase-1 plays a role in bacterial killing independent of the NLRP3 inflammasome complex.

As nigericin promotes potassium efflux, and potassium is involved in the promotion of phagocytosis (31), we sought to determine if the effects on bacterial killing were the result of increased host cell death and found this not to be the case. Our results demonstrated that nigericin did not alter *C. rodentium* growth rates directly (Figure 3A) and did not result in statistically significant changes in phagocytosis in Raw 264.7 cells (Figures 3B,C). To identify key targets involved in response to nigericin we ran an inflammasome gene array of Raw 264.7 cells treated with nigericin and compared expression of these genes to untreated (NT) cells (Figure 4). Validation of expression of key target genes demonstrated that nigericin resulted in increased expression of Cd40lg, Ciita (NLRA), IL-12, IFNγ, Mapk11 (p38), Nlrp4e, and Tab2 as well as a decrease in Tnfsf11 in nigericin-treated cells (Figure 5). Based on these results we hypothesized a possible model of nigericin-mediated bacterial killing without involvement of the inflammasome (Figure 6). Caspase-1 has previously been shown to be modulated by nigericin via interactions with cathepsin B (32). Interestingly, caspase-1 promotes cleavage of the pro-inflammatory cytokine pro-IL-18 to active IL-18. Activation of IL-18 leads to up-regulation of Cd40lg (33), a cytokine which was increased in response to nigericin in the current study. CD40 is thought to play a key role in inflammatory diseases as hyperactivation of CD40 leads to increased production of pro-inflammatory cytokines, including IL-12 (34). IL-12 was also found to be increased in response to nigericin. Results from the current study support previous research indicating that both IL-12 and IL-18 promote production of IFNγ. This cytokine plays a vital role in induction of the MHC class II complex, which is critical in macrophage defense against viral, protozoal, and some bacterial infections (35). IFNγ is an upstream activator of both MAPK and ciita, also known as NLRA. Both p38 and ciita were increased following nigericin treatment, adding further support for our hypothesized pathway. The Ciita/STAT1 pathway is important for MHCII activation and antigen presentation following phagocytosis of bacteria in macrophages (36), while IFNγ induction of p38 promotes phagocyte and bacterial killing pathways via oxidative burst by increased ROS production (37, 38). Interestingly, we support this model by demonstrating that while IL-1β secretion was not effected in Raw 264.7 or J77^ASC−/−^ cells, secretion of a closely related interleukin, IL-18, was increased in these cells in response to nigericin (Figure 2). We further showed that phosphorylated p38 was increased in these cells in response to nigericin indicating increased activity (Figure 7). Knockdown of the key pathway intermediates p38, IFNγ, or both, results in reduced bacterial killing only in cells lacking ASC (Figure 8), indicating this pathway is indeed involved as an alternate mechanisms of bacterial killing to NLRP3/ASC inflammasome complex pathways. This work should stimulate further investigation into alternative, non-inflammasome mediated pathways for immune activation and bacterial killing in macrophages.

We have demonstrated here the potential for pathways alternate to the classic NLRP3 inflammasome in bacterial killing in macrophage cells lacking an active inflammasome complex by utilizing nigericin *in vitro*. Macrophages play a key role in clearance of pathogenic and pathobiont microorganisms in inflammatory disease (39). Therefore, this study has the potential to impact a number of inflammatory diseases including cystic fibrosis, inflammatory bowel diseases, infection, and even cancer, as this could introduce innovative treatment options to mitigate disease pathogenesis by targeting multiple immune pathways to improve efficiency of bacterial clearance (40). As current studies are focused on targeting the NLRP3 inflammasome alone in these diseases, understanding the alternate inflammatory pathways involved in bacterial clearance within a macrophage cell system will aid in our ability to identify new methods of modulating immune response.

## Author Contributions

HA, MB-J, and EW conceived and designed the experiments. HA, MB-J, and RC performed the experiments. HA and MB-J were responsible for figures preparation and statistical analyses. HA and EW drafted the manuscript. All authors approved the final version.

### Conflict of Interest

The authors declare that the research was conducted in the absence of any commercial or financial relationships that could be construed as a potential conflict of interest.

## Supplementary Material

Supplementary Figure 1 Growth response of Raw 264.7 cells to the indicated doses of ATP, YVAD, nigericin, and gentamicinin was examined by manual counting using haemocytometer at indicated times and by trypan blue exclusion. Cell growth (cell/mL) was interpreted by examining live cell counts only at each time point; % death represents the percentage of dead cells counted by trypan blue exclusion as a percentage of total cells.

Supplementary Figure 2 Growth rate of *C. rodentium* in **(A)** LB medium or **(B)** following gentamicin treatment for indicated time points where time 0 h contained 10^∧^8 CFU/mL. Turbidity was determined by serial dilutions of an overnight LB culture of *C. rodentium* plated and grown on LB agar overnight. Manual colony counts were performed on the serially diluted cultures grown on LB agar plates to determine CFU/mL.

Supplementary Figure 3 Intracellular IL-1β and IL-18 are not altered in response to infection or drug treatment in Raw264.7 or J77 cells. Raw264.7 cells or control J77 cells were treated as indicated and inoculated with *C. rodentium* (MOI = 1:10). ELISA of cytosolic lysates of J77 and Raw264.7 cells confirm intracellular expression of **(A)** IL-1β and **(B)** IL-18. Samples were compared to non-infected control (NI) cells which received no treatment or infection. Values represent mean±SEM between 3 independent experiments. YVAD (Ac-Tyr-Val-Ala-Asp-Chloromethylketone); MOI (multiplicity of infection).

Supplementary Figure 4 QIAGEN inflammasome gene array was performed following the manufacturer's instructions on RNA collected from Raw 264.7 cells treated with nigericin and was compared to untreated cells, following C. rodentium infection. **(A)** Fold change (treated/untreated) results are displayed as a heat map with **(B)** gene layout and associated fold change values. ‘A’ signifies a statistically significant difference between control and nigericin treatment; ‘B’ signifies *p* > 0.05; ‘C’ signifies the expression of this gene was undetectable.
