# Supplementary figures and images for: Nigericin Promotes NLRP3-Independent Bacterial Killing in Macrophages
Source: Front Immunol. 2019 Oct 1;10:2296. doi: 10.3389/fimmu.2019.02296 (PMC6779719; doi:10.3389/fimmu.2019.02296)

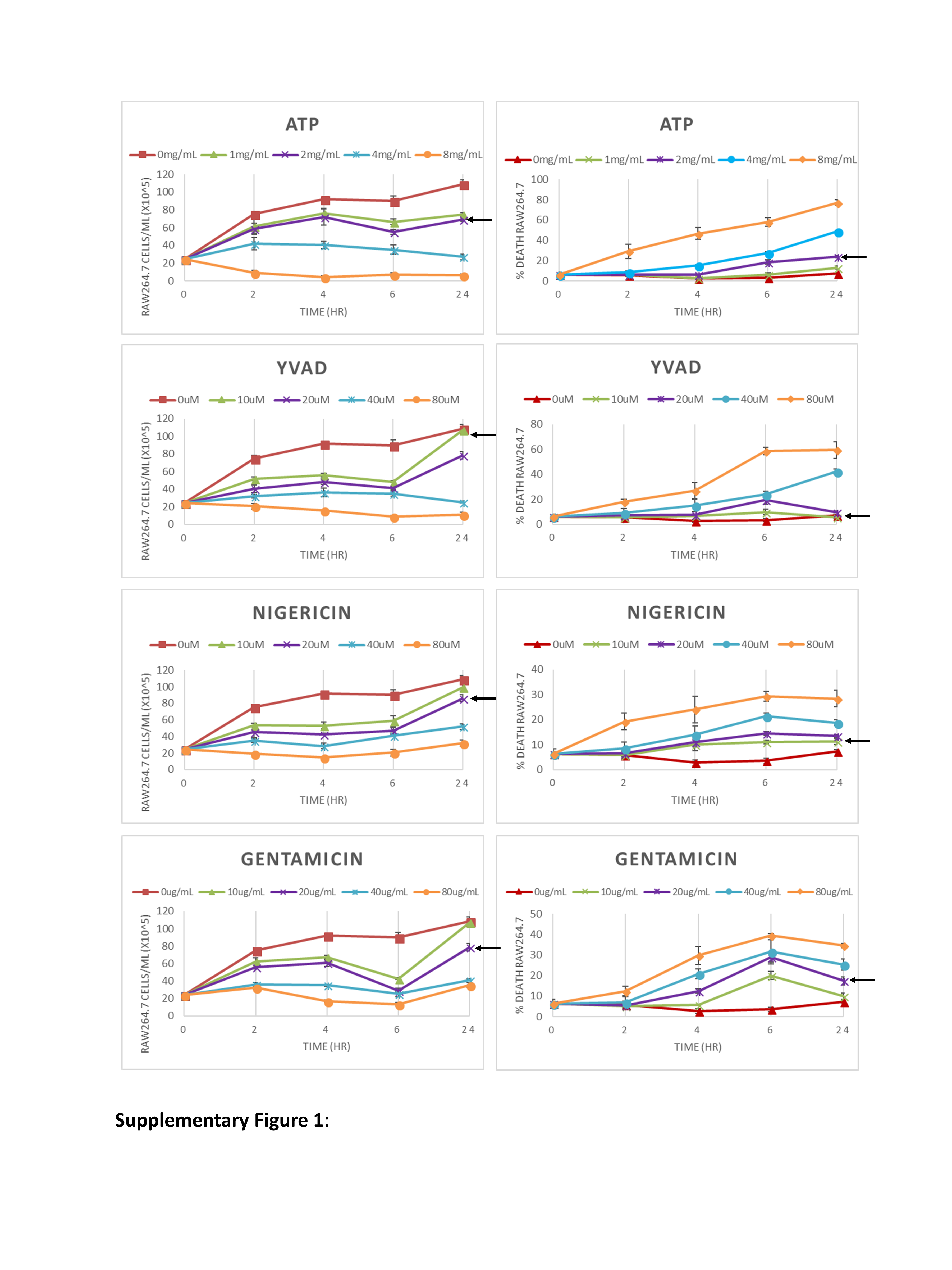

Supplement: Supplementary Figure 1 — Growth response of Raw 264.7 cells to the indicated doses of ATP, YVAD, nigericin, and gentamicinin was examined by manual counting using haemocytometer at indicated times and by trypan blue exclusion. Cell growth (cell/mL) was interpreted by examining live cell counts only at each time point; % death represents the percentage of dead cells counted by trypan blue exclusion as a percentage of total cells. [file Image_1.TIF]

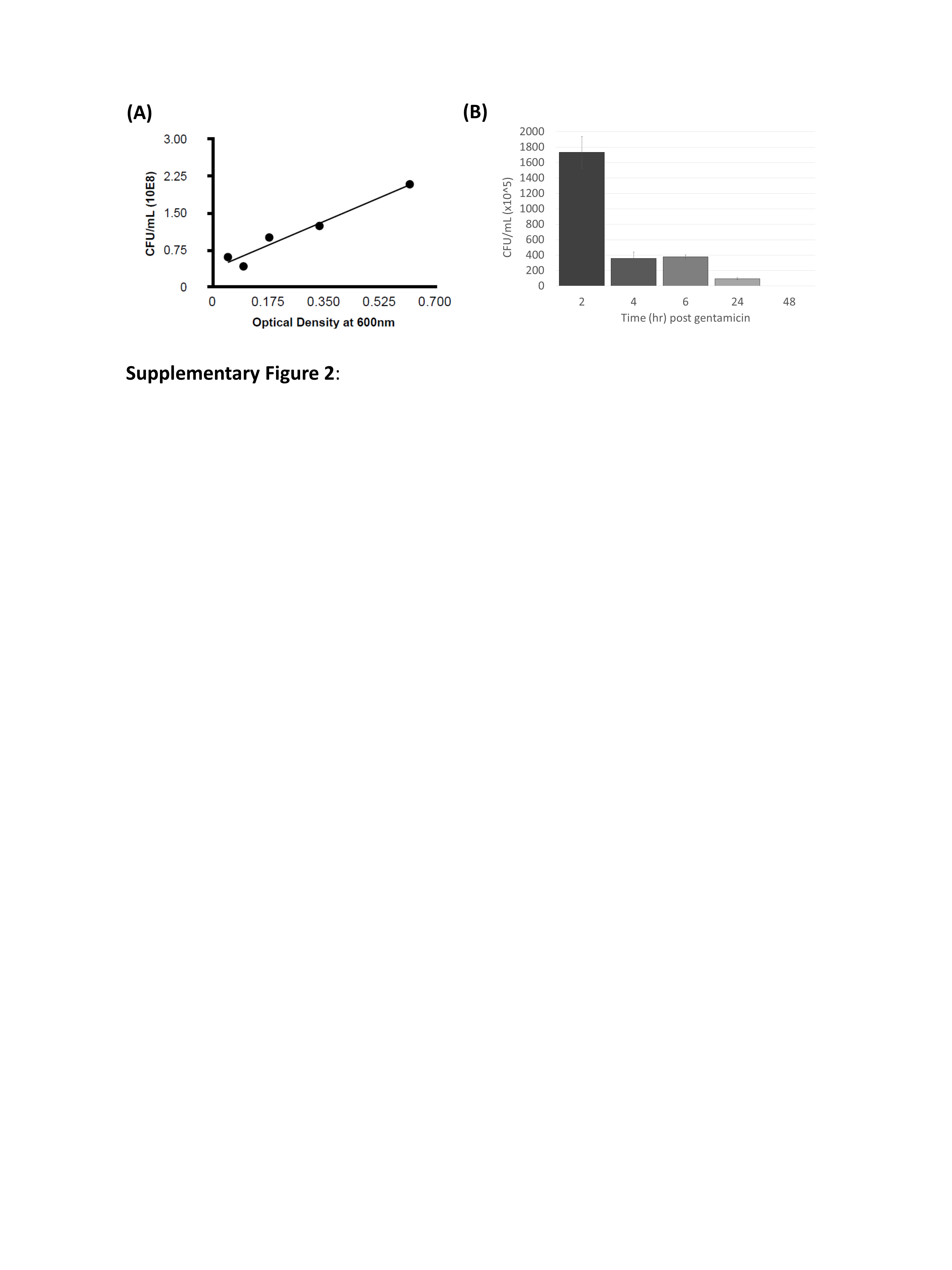

Supplement: Supplementary Figure 2 — Growth rate of C. rodentium in (A) LB medium or (B) following gentamicin treatment for indicated time points where time 0 h contained 10∧8 CFU/mL. Turbidity was determined by serial dilutions of an overnight LB culture of C. rodentium plated and grown on LB agar overnight. Manual colony counts were performed on the serially diluted cultures grown on LB agar plates to determine CFU/mL. [file Image_2.TIF]

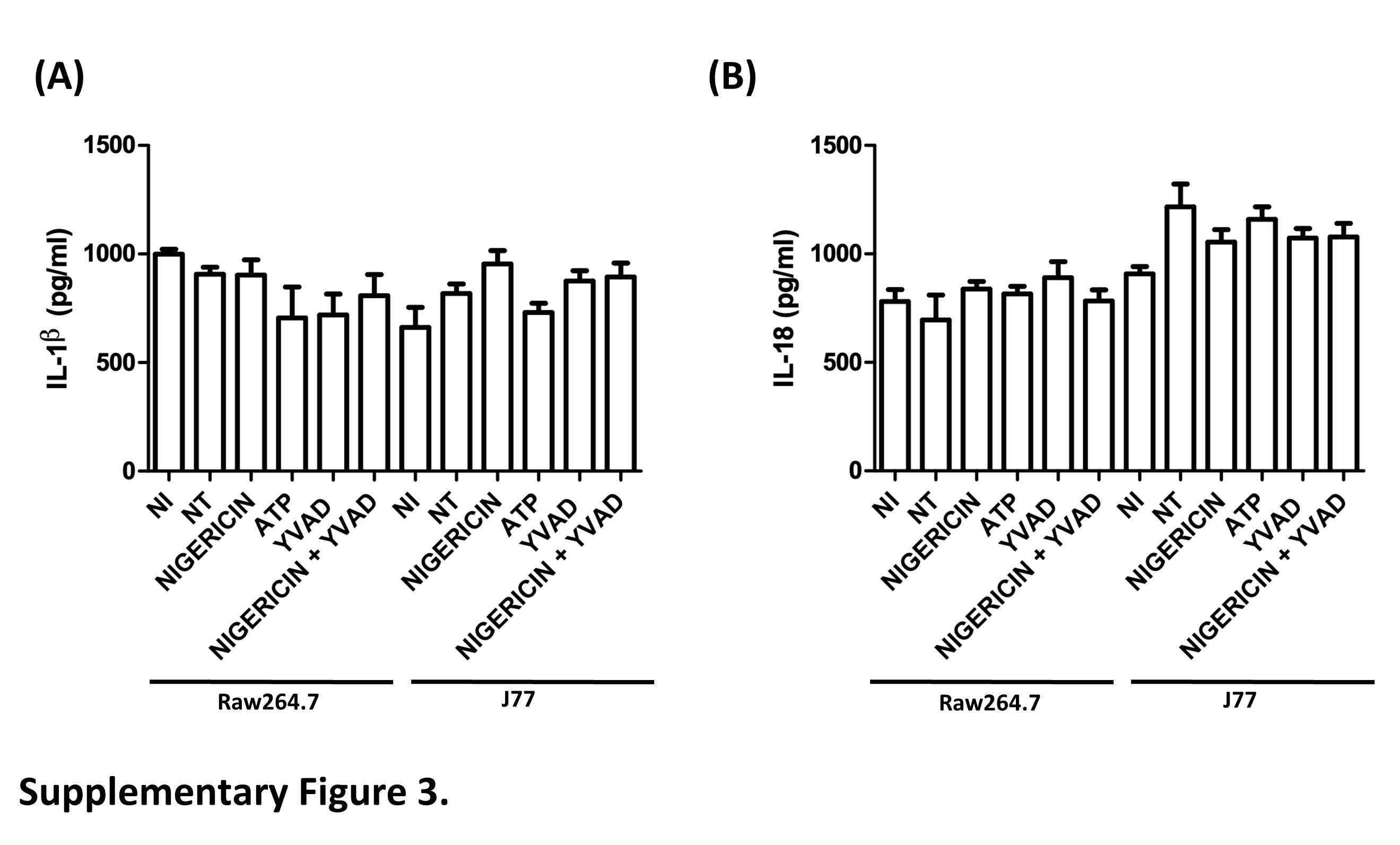

Supplement: Supplementary Figure 3 — Intracellular IL-1β and IL-18 are not altered in response to infection or drug treatment in Raw264.7 or J77 cells. Raw264.7 cells or control J77 cells were treated as indicated and inoculated with C. rodentium (MOI = 1:10). ELISA of cytosolic lysates of J77 and Raw264.7 cells confirm intracellular expression of (A) IL-1β and (B) IL-18. Samples were compared to non-infected control (NI) cells which received no treatment or infection. Values represent mean±SEM between 3 independent experiments. YVAD (Ac-Tyr-Val-Ala-Asp-Chloromethylketone); MOI (multiplicity of infection). [file Image_3.TIF]

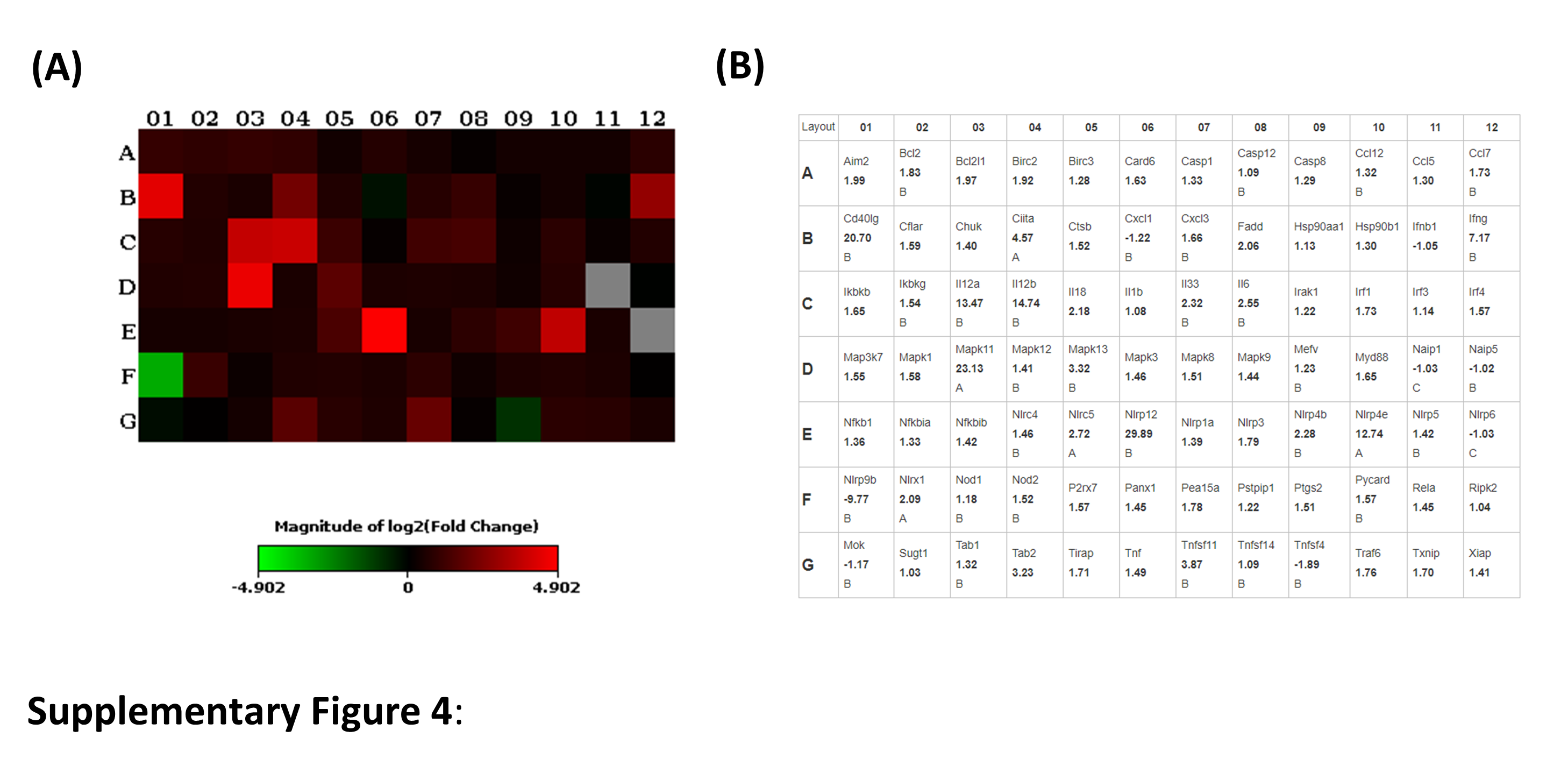

Supplement: Supplementary Figure 4 — QIAGEN inflammasome gene array was performed following the manufacturer's instructions on RNA collected from Raw 264.7 cells treated with nigericin and was compared to untreated cells, following C. rodentium infection. (A) Fold change (treated/untreated) results are displayed as a heat map with (B) gene layout and associated fold change values. ‘A’ signifies a statistically significant difference between control and nigericin treatment; ‘B’ signifies p > 0.05; ‘C’ signifies the expression of this gene was undetectable. [file Image_4.tif]
